# Immunolocalization of G protein-coupled estrogen receptor in the rat epididymis

**DOI:** 10.1186/s12958-015-0042-z

**Published:** 2015-05-27

**Authors:** Griselle B. Martínez-Traverso, Christopher A. Pearl

**Affiliations:** Department of Biological Sciences, Western Michigan University, Kalamazoo, Michigan USA; Departments of Biology and Industrial Microbiology, University of Puerto Rico, Mayagüez, Puerto Rico

**Keywords:** Estrogen receptor, Estrogen, Epididymis, GPER, Rat

## Abstract

**Background:**

Estrogen plays an important role in male reproduction, and males lacking estrogen signaling in the reproductive tissues are infertile. Estrogen signaling is mediated via two nuclear receptors, ERα and ERβ, but it was recently found that a G protein-coupled estrogen receptor (GPER) is present in the testis. It is believed that GPER is a membrane form of the estrogen receptor and mediates non-classical estrogen signaling. However, the cellular localization of GPER in the epididymis is unknown. Therefore, the objective of this study was to determine the cellular and regional expression of GPER in the rat epididymis.

**Findings:**

To localize expression, immunohistochemistry (IHC) was performed using fixed epididymal tissue. Three strains and ages of rats were used to identify whether GPER expression is strain or age specific. Our results are the first to demonstrate immunostaining of GPER in epididymal epithelial cells. Expression was highest near the apical membrane followed by the cytoplasm, consistent with a membrane bound receptor. The highest expression in adult rats was observed in corpus followed by cauda. Western blotting analysis of epididymal tissues from Sprague Dawley rats confirmed specificity of the antibody and regional expression.

**Conclusions:**

Expression of GPER in the corpus and cauda suggests a role for non-classical estrogen signaling in sperm maturation in the corpus, and sperm protection/storage in the cauda. GPER expression pre-pubertally suggests that estrogen may have a role in epithelial cell development in addition to regulation of adult function.

## Findings

### Background

The epididymis serves as the link between the efferent ductules and the vas deferens in order for sperm to exit the testis. Transit through the epididymis is necessary for spermatozoa to completely mature, become motile and acquire fertility. This process can take up to two weeks in order for spermatozoa to travel through the entire epididymis. The principal function of the epididymis is regulating the luminal milieu to provide the proper environment for sperm maturation [1]. In addition to sperm maturation and sperm transport, the epididymis stores sperm prior to ejaculation [2].

The epididymis is regulated by androgen and estrogen signaling via androgen receptor (AR), or by the two nuclear estrogen receptors: estrogen receptor alpha (ERα) and estrogen receptor beta (ERβ). AR is present in the nuclei of all epididymal regions and ERβ is present in the nuclei and cytoplasm of all epididymal regions in rats and other mammalian species [3–5]. ERα is present in epididymal epithelial cell nuclei and cytoplasm, and on the sperm in rats [3].

A third form of the estrogen receptor, G protein-coupled estrogen receptor (GPER), has been identified. GPER is expressed by immature Sertoli cells, pachytene spermatocytes and round spermatids in the rat testis [6–8]. GPER has been reported to be present in the epididymis based on mRNA and western blot analysis [9, 10] but GPER immunolocalization by region and cell type is still unknown. To better understand the role of GPER in epididymal function, this study was designed to determine the cellular and regional expression of GPER and investigate any difference between ages and strains of rats.

## Methods

### Animals and tissue collection

Sprague Dawley, Brown Norway and Wistar rats were housed with free-will access to food and water until the desired age was reached. Adult, sexually mature Brown Norway (n = 4), Wistar (n = 3) and Sprague Dawley (n = 3) rats were used to investigate potential strain differences in GPER expression. After euthanasia by CO_2_, epididymal tissue was dissected and fixed in 4 % paraformaldehyde solution. Tissue was serially dehydrated and stored in 70 % ethanol. Additionally, to investigate potential age differences, tissues were collected from three Sprague Dawley rats at 4- and 8-weeks of age. Tissue was embedded in paraffin wax and cut in 5 μm slices that were mounted on microscope slides for immunohistochemistry. All procedures had approval from the Western Michigan University Institutional Animal Care and Use Committee.

### Immunohistochemistry (IHC)

The localization of GPER in the initial segment (IS), caput, corpus and cauda tissues was investigated by immunohistochemistry using a protocol similar to one previously described [3]. Tissue was paraffin embedded and sectioned at a thickness of 5 μm. Antigen retrieval was performed by submerging slides in a citric acid based antigen unmasking solution (Vector Laboratories, Inc., Burlingame CA) in Coplin jars and steam heated to 93 °C for 5 min after which they were allowed to cool to room temperature. Endogenous peroxidase activity was blocked by incubation in 0.3 % hydrogen peroxide in methanol for 30 minutes. After a blocking step, tissues were incubated for two hours at room temperature with rabbit anti-human GPR30 (1:2500; sc-48525; Santa Cruz Biotechnology). Following primary antibody incubation, sections were incubated with goat anti-rabbit biotinylated secondary antibody followed by an avidin–biotin horseradish peroxidase complex (Vector Laboratories). Immunostaining was visualized using NovaRed chromagen (Vector Laboratories). Pre-absorbed primary antibodies were substituted for primary antibody as a negative control; all other steps were identical including exposure times with NovaRed. All slides were counterstained with Immunomaster Hematoxylin and evaluated by light microscopy.

### Imaging

Slides/tissues were visualized using a Nikon 80i microscope and images were taken with NIS Elements Software. Acquisition of images was done using a Nikon Digital Sight camera with NIS Elements Software. Intensity of immunostaining was scored as negative (−), weak positive (+), moderately positive (++) or strong positive (+++) by two independent observers.

### Western blotting

Initial segment/caput, corpus and cauda were homogenized in a buffer consisting of 50 mM Tris-Base, 10 mM EDTA, 150 mM NaCl, 0.1 % Tween 20 at pH 7.6. Homogenates were separated on a SDS-polyacrylamide gel with a MiniProtean Tetra System (BioRad; Hercules, CA, USA) using Precision Plus Protein molecular weight standards (BioRad) and transferred to an Immobilon-FL membrane (Millipore, Bedford, MA, USA). Membranes were blocked with 5 % nonfat dry milk for one hour at room temperature. GPER antibody (1:250 in 5 % nonfat dry milk) was incubated overnight at 4 °C. Pre-absorbed primary antibody and actin antibody were also incubated overnight at 4 °C. Membranes were washed and incubated with alkaline phosphatase-conjugated goat anti-rabbit IgG (Applied Biosystems) diluted 1:10,000 in 5 % nonfat dry milk for one hour at room temperature. Membranes were visualized with chemiluminescence (Tropix CSPD with Nitro Block; Applied Biosystems) and exposed on Blue Basic AutoRad film (Bioexpress; Kaysville, UT, USA).

## Results

**Fig. 1:**
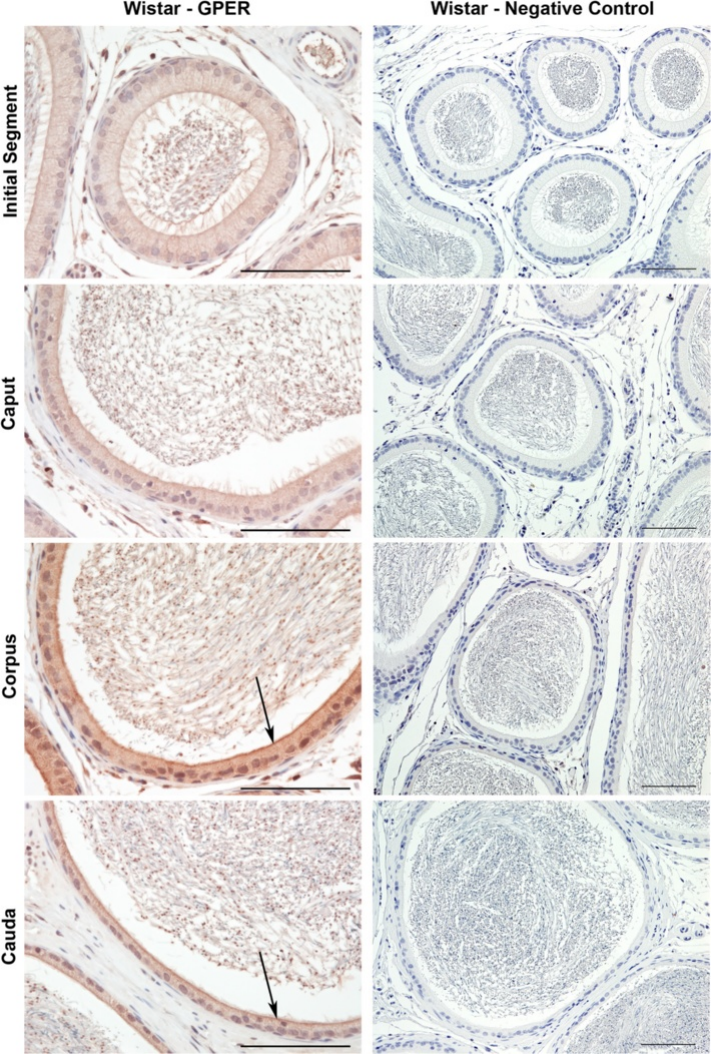
Immunolocalization of GPER in adult Wistar rats. GPER immunostaining is observed primarily in the principal cell cytoplasm (red color) within the corpus and cauda. Immunostaining is strongest near the apical membrane (arrows). No immunostaining was observed when slides were incubated with pre-absorbed primary antibody. Bar = 100 μm

**Fig. 2:**
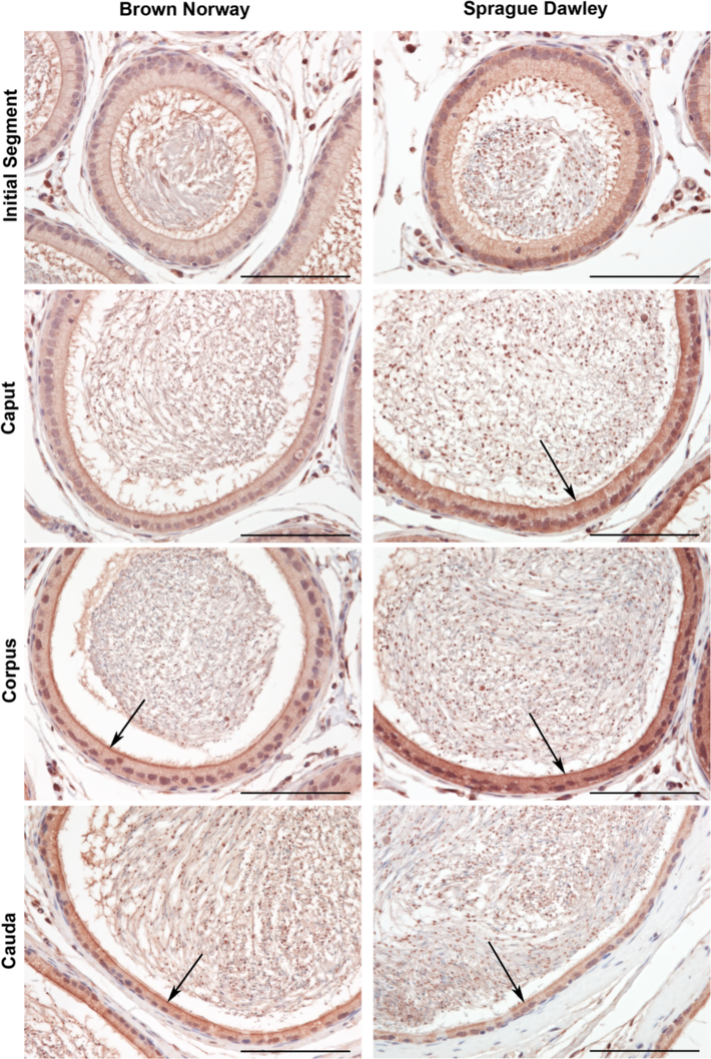
Immunolocalization of GPER in adult Brown Norway and Sprague Dawley rats. GPER immunostaining is observed primarily in the principal cell cytoplasm (red color) within the corpus and cauda. Immunostaining is strongest near the apical membrane (arrows). Bar = 100 μm

**Fig. 3:**
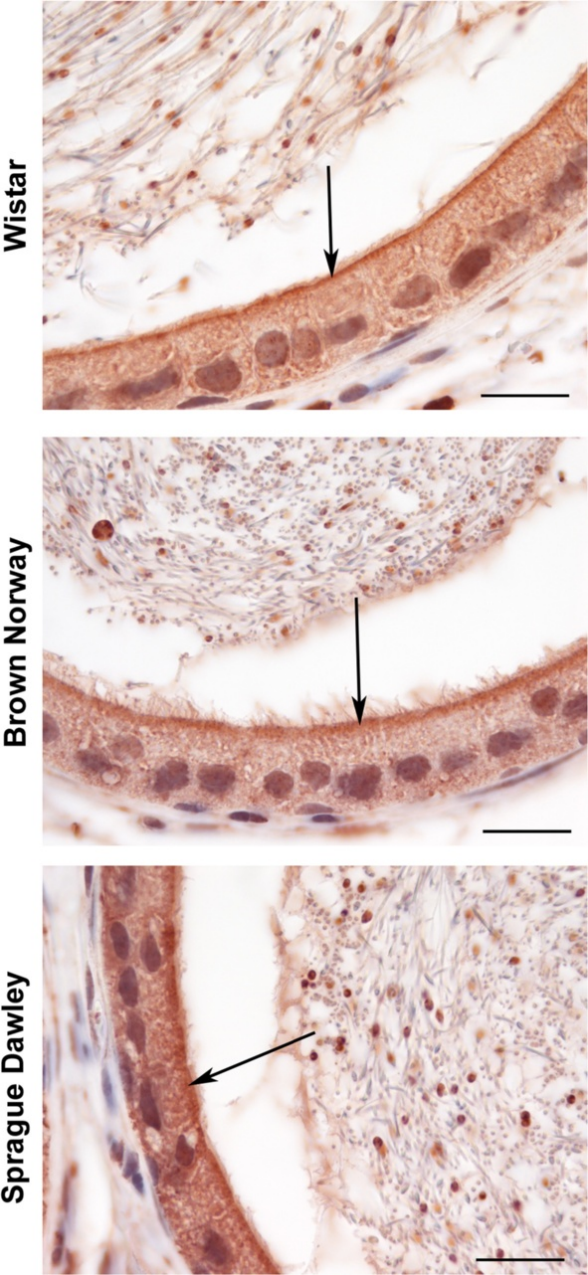
Immunolocalization of GPER in the corpus of adult Wistar, Brown Norway and Sprague Dawley rats at higher magnification. GPER immunostaining is observed in the cytoplasm (red color) and immunostaining is strongest near the apical membrane (arrows). Bar = 20 μm

**Fig. 4:**
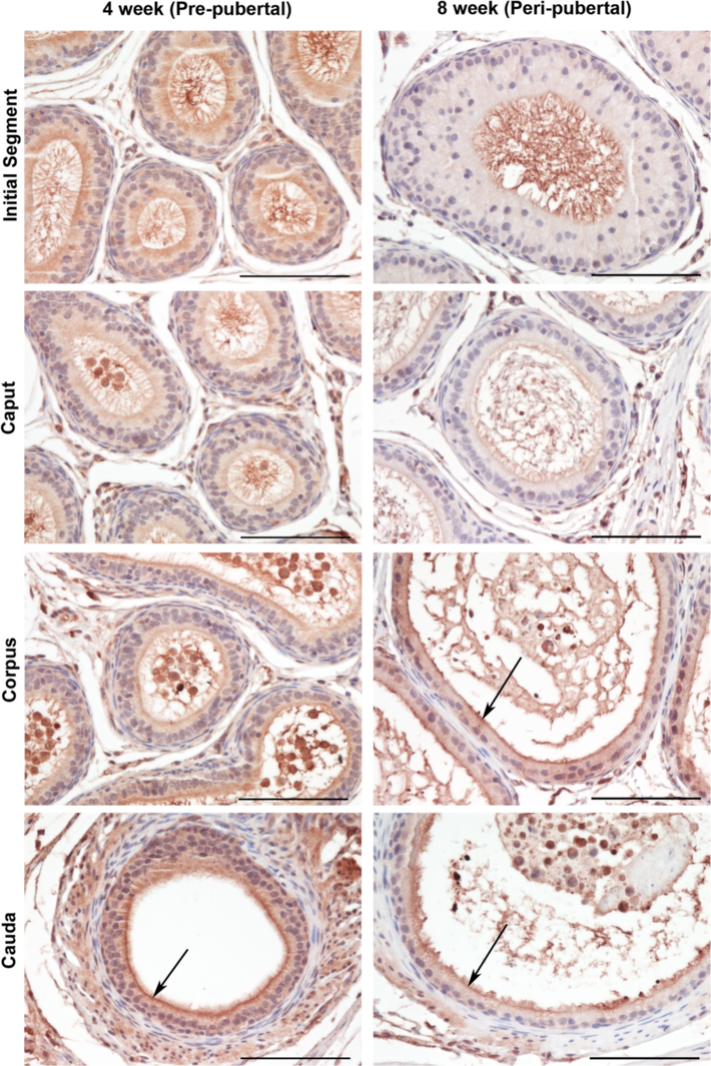
Immunolocalization of GPER in four week old pre-pubertal and eight week old peri-pubertal Sprague Dawley rats. In pre-pubertal rats, GPER immunostaining is observed primarily in the epithelial cell cytoplasm (red color). Immunostaining is strongest in the cauda and near the apical membrane (arrows). In peri-pubertal rats, GPER immunostaining is observed primarily in the epithelial/principal cell cytoplasm within the corpus and cauda. Immunostaining is strongest near the apical membrane (arrows). Bar = 100 μm

GPER immunostaining was observed primarily in the principal cell cytoplasm of adult Wistar (Fig. 1), Brown Norway (Fig. 2) and Sprague Dawley (Fig. 2) rats. In the cytoplasm, the most intense immunostaining was observed near the luminal/apical membrane giving the appearance of an apical ring. Immunostaining in all three strains was highest in the corpus followed by the cauda (Fig. 3; Table 1). The initial segment and caput displayed weak immunostaining in Wistar and Brown Norway rats while these regions were moderately positive in Sprague Dawley rats (Table 1). In contrast to ERα and ERβ which display strong nuclear staining [3], nuclei in the corpus and cauda of adult animals were weakly positive for GPER immunostaining. In four week old pre-pubertal Sprague Dawley rats, GPER immunostaining was strongest in the cauda, while epithelial cytoplasm was also positive in the initial segment, caput and corpus (Fig. 4). In eight week old peri-pubertal Sprague Dawley rats, the immunostaining pattern was similar to that observed in adult rats (Fig. 4). Tissues incubated with pre-absorbed primary antibody were negative for GPER immunostaining (Fig. 1).

**Table 1:** Summary of GPER immunostaining in the epididymis of adult Sprague Dawley (SD), Brown Norway (BN) and Wistar rats

Region	Rat Strain	Cytoplasm	Apical Surface	Nuclei
Initial Segment	Adult SD	++	+/−	-
	Adult BN	+	+/−	-
	Adult Wistar	+	+/−	-
Caput	Adult SD	++	+++	-
	Adult BN	+	++	-
	Adult Wistar	+	+	-
Corpus	Adult SD	+++	+++	+
	Adult BN	++	+++	+
	Adult Wistar	++	+++	+
Cauda	Adult SD	++	++	+/−
	Adult BN	++	+++	+/−
	Adult Wistar	+	++	+

Intensity of immunostaining was scored as negative (−), weak positive (+), moderate positive (++) or strong positive (+++)

**Fig. 5:**
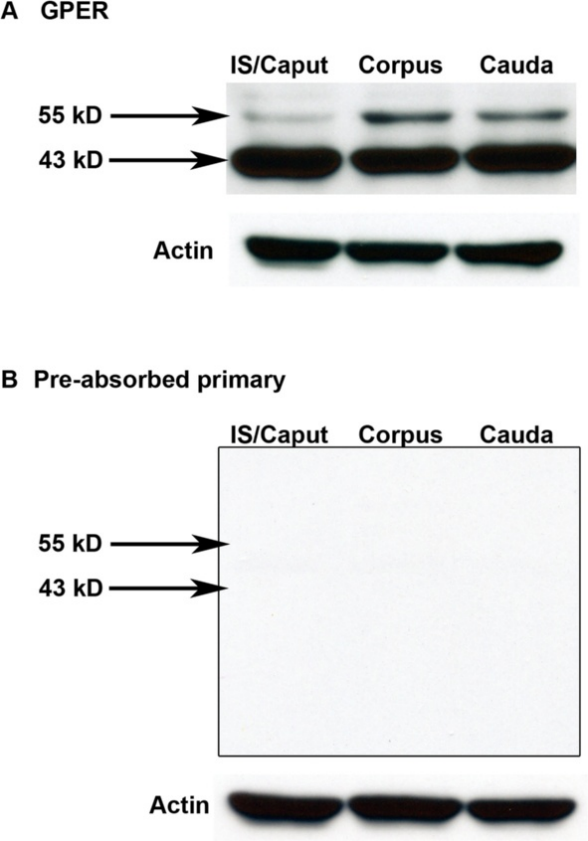
Western blot for GPER in the IS/caput, corpus and cauda from Sprague Dawley rats. **a** Proteins of appropriate molecular weight for GPER were observed at 43 kD and 55 kD. Equal amounts of protein were loaded into each lane which was confirmed by actin immunoblotting. **b** No protein bands were observed when membranes were incubated with pre-absorbed primary antibody. Images shown are representative of results from three separate animals

Using western blot analysis, a primary protein band was observed at 43 kD and a secondary band was observed at 55 kD in epididymal tissues from Sprague Dawley rats (Fig. 5). GPER has been reported as a 43 kD protein in the rat testis [7, 8] and a 55 kD protein in the epididymis of Wistar rats [10] and our antibody appears to detect both forms. Expression of the 43 kD protein appeared similar between epididymal regions while expression of the 55 kD band appeared stronger in the corpus and cauda compared with the IS/caput. The western blot results as currently performed were not designed to confirm the subcellular localization of GPER, however, they do confirm antibody specificity and provide support for immunohistochemistry results suggesting lower GPER expression in the initial segment/caput.

## Discussion

Our results demonstrate the cellular localization and regional expression pattern of GPER in the epididymis of multiple strains of rats and at various developmental stages. In adult animals, GPER was expressed primarily in the corpus and cauda with lower expression observed in the initial segment and caput. Our results are consistent with previous reports measuring GPER protein via western blotting [10] and mRNA [9] that observed the highest expression in the corpus of Wistar rats. The expression pattern of GPER was quite similar in Wistar and Brown Norway rats. Sprague Dawley rats show a broader expression since the caput and initial segment have a stronger immunostaining in these regions compared to the other strains examined. ERα and ERβ exhibit nuclear expression and function as classical nuclear receptors and transcription factors. GPER expression was primarily cytoplasmic suggesting that estrogen action via GPER may be a separate signaling pathway from the nuclear receptors ERα and ERβ. Expression of GPER is highest in the corpus, but interestingly, concentrations of estradiol in the corpus of Wistar rats have been reported be significantly lower than the IS/caput and cauda [10].

In the rat testis, GPER is expressed by pachytene spermatocytes, round spermatids and Sertoli cells where it appears to function in cell differentiation and regulation of apoptosis [6–8]. While the function of GPER in the epididymis remains to be determined, it may be related to the regulation of regional gene expression. Epididymal gene expression is region specific with genes such as beta-defensins and lipocalins showing higher expression in the corpus [11–13]. Proteins such as lactoferrin and clusterin also show higher expression in the corpus, compared to other epididymal regions [14, 15]. In general, the percentage of mature sperm greatly increases in the corpus where GPER expression is highest, which suggests GPER signaling may have a role in sperm maturation. Also, since GPER is expressed in the cauda where sperm are stored, GPER signaling may be important for sperm protection and storage. The presence of GPER in pre-pubertal rats suggests that the estrogen signaling via GPER may have a function in epithelial cell development. GPER expression in peri-pubertal rats suggests that the estrogen signaling via GPER may also play a role in epithelial differentiation and maturation.

In conclusion, our results suggest that non-classical estrogen signaling via GPER may exist in the epididymis and contribute to sperm maturation in the corpus and storage in the cauda.
